# A Systemic Network Triggered by Human Cytomegalovirus Entry

**DOI:** 10.1155/2011/262080

**Published:** 2011-05-15

**Authors:** Anyou Wang, Li Ren, Hong Li

**Affiliations:** ^1^School of Public Health, University of California, Berkeley, CA 94720, USA; ^2^Department of Human Genetics, David Geffen School of Medicine, University of California Los Angeles, Los Angeles, CA 90095-7088, USA; ^3^Center for Clinical Investigation, School of Medicine, Stanford University, Stanford, CA 94305, USA

## Abstract

Virus entry is a multistep process that triggers various cellular pathways that interconnect into a complex network; yet the molecular complexity of this network remains largely elusive. Here, by employing systems biology approaches, we reveal a systemic virus-entry network initiated by human cytomegalovirus (HCMV), a widespread opportunistic pathogen. This network contains ten functional modules (i.e., groups of proteins) that coordinately respond to HCMV entry. Functional modules activated (up- and downregulated) in this network dramatically decline shortly within 25 minutes post infection. While modules annotated as receptor system, ion transport, and immune response are continuously activated during the entire process of HCMV entry, those annotated for cell adhesion and skeletal movement are specifically activated during viral early attachment. The up-regulated network contains various functional modules, such as cell surface receptors, skeletal development, endocytosis, ion transport, and chromatin remodeling. Interestingly, macromolecule metabolism and chromatin remodeling module predominates this over-expressed system, suggesting that the fundamental nuclear process modulation is one of the most important events in HCMV entry. The entire up-regulated network is primarily controlled by multiple elements like SLC10A1. Thus, virus entry triggers multiple cellular processes especially nuclear processes to facilitate its entry.

## 1. Introduction

For decades, intensive studies on individual genes and pathways involved in virus entry have successfully provided us with an unprecedented wealth of molecular detail on how component proteins respond to virus entry [1]. Quite unexpectedly, however, drugs targeting individual components of specialized pathways identified in single-component studies not only failed to control virus infection but also caused huge unexpected side-effects [2]. Clearly, virus entry is not simply the result of a single activated gene or pathway but a complex network of various cellular pathways and its components. Many proteins and pathways are continuously cross-talking to coordinate cellular signals during each step of virus entry, such as virus attachment, interaction with receptors, signaling, membrane fusion, and endocytosis. Nevertheless, the global picture on how these proteins interact with each other to permit virus entry into cells remains incomplete. In particular, very little is known about the systemwide network and functional modules involving virus entry. This type of knowledge is the initial step towards completely elucidating the complexity of virus entry and developing efficient treatments to prevent virus spread to other cells. 

Human cytomegalovirus (HCMV) is a ubiquitous opportunistic pathogen with diverse genomes [3] that causes fatal or permanently debilitating disease in immunologically compromised individuals and neonates. Particularly at risk for infection with this virus are AIDS patients, cancer patients, organ or tissue transplant recipients undergoing immunosuppressive therapy, infants, fetuses, and the elderly. More recently, the virus has also been implicated in tumorigenesis [4], and the etiology of circulatory diseases, most notably, atherosclerosis [5]. 

HCMV entry into cells activates (up- and downregulates) a variety of signaling pathways and multiple cellular receptors. HCMV attachment/entry during 5 to 25 min post infection (PI) triggers components and pathways linked to receptor tyrosine kinase, mitogen-activated protein (MAP) kinase signaling, cytoskeletal rearrangement, transcription factors, prostaglandins, and cytokines [6]. In particular, HCMV entry activates epidermal growth factor receptor (EGFR), avb3 integrin (a2ß1, a6ß1, and avß3), platelet-derived growth factor receptor-alpha (PDGFR*α*), and their signaling pathways [7–12], which play important roles in HCMV entry. Therefore, EGFR, PDGFR*α*m, and avb3 integrin have been proposed as HCMV receptors [7–12]. However, EGFR is not expressed on all HCMV-permissive cell types that are efficiently infected by HCMV. In addition, EGFR might not be essential for all HCMV entry [8]. Integrins likely play a role in downstream events during HCMV entry [7–10]. The extent of conservative role of PDGFR*α* in HCMV entry remains to be characterized. In vivo, HCMV can infect almost every organ system and tissue type [6, 8, 13], and in vitro HCMV can promiscuously penetrates diverse cell lines with varying receptors. Together, these findings suggest that HCMV entry activates multiple proteins interacting into a network that remains largely elusive. Elucidating such an HCMV entry network could provide valuable insights into the mechanism of virus entry in general.

In this study, we used systems biology approaches as we previously reported [14] to systematically elucidate a comprehensive systemic network triggered by HCMV entry. Our work provides a conceptual framework to further understand the fundamental molecular basis of virus entry.

## 2. Results

### 2.1. A Comprehensive Protein-Interaction Network Linked to HCMV Entry

**Figure 1:**
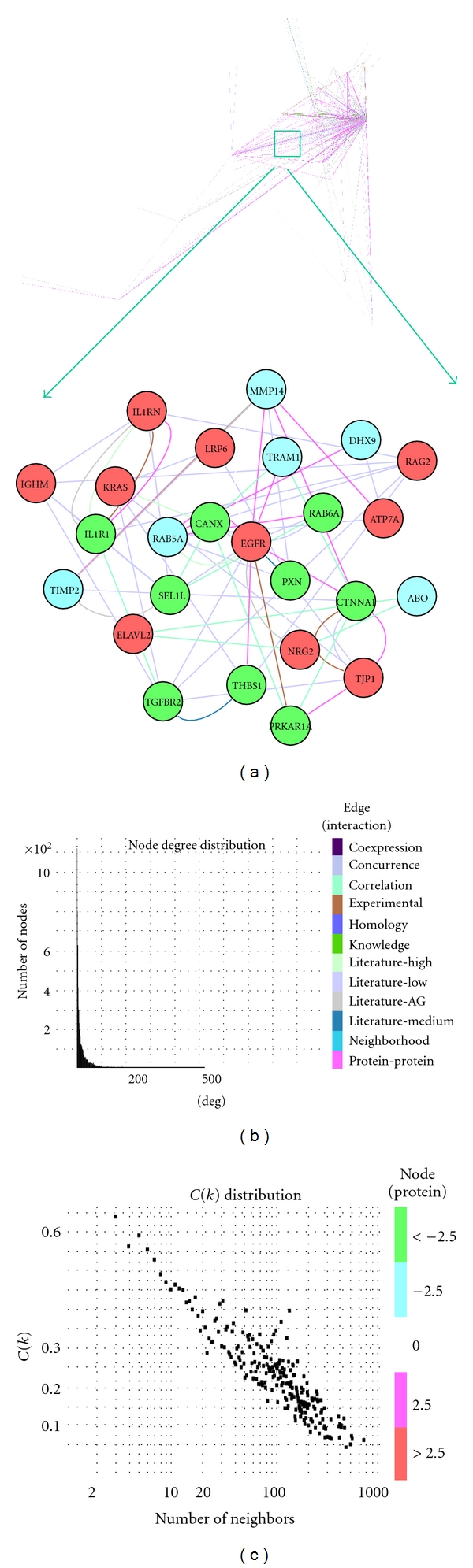
A comprehensive regulatory network linked to HCMV entry. The network was constructed by using protein-protein binding database and protein functional database (see text and Section 4 for details). The insert shows a zoomed portion of entire network. The colors of nodes (proteins) and edges (interactions) represent gene expression levels (red color-upregulation, green color-downregulation compared with mock-control) and edge sources, respectively. The same color strategy for nodes and edges will be used for all figures in this study unless otherwise specified. Also shown are entire network properties, including node degree distribution that approximates a power law, *P*(*k*) ~ *k* ^−*γ*^ (*γ* = 0.95 in our network), and *C*(*k*) distribution, average clustering coefficient that measures the tendency of nodes to form clusters, which decreases with the number of neighbors.

To systematically decode the systemic network activated by HCMV entry, we first utilized systems network approaches expended from our previous report [14] to search published databases for human physical and functional protein-protein interactions known to date (Section 4). These interactions were then combined into a systemic protein-interaction network database, which currently comprises 6651 nodes (proteins) and 64392 edges (interactions) (Figure 1(a), see Table S1 in Supplementary Material available online at doi:10.1155/2011/262080). The interactions (edges) include 12 types of interactions. For example, coexpression represents that source gene and targeted gene have coexpression relationship extracted from database (see Section 4 for database we used in this study). 

To examine the overall architectural features of this network, we analyzed overall node degree distribution, which represents the possibility of nodes having a given degree, and the number of incident edges to a given node. The node degree distribution of our network decreases with degree and approximates a power law (Figure 1(b)), indicating that our network is a scale-free network, which is proposed as a universal network framework in biology networks [15–17]. In addition, we also calculated the average of the clustering coefficient *C*(*k*) distribution, which describes how nodes link to others via their *K* neighbor to form clusters or groups. *C*(*k*) also diminishes with the increase in number of neighbors (Figure 1(b)), indicating that our network is a hierarchical network [15–17] predominated by hubs (highly connected proteins) and bottlenecks, which are nodes with many shortest paths going through them analogous to key bridges that link subnetworks to a whole map network [18]. Both hubs and bottlenecks are likely to play essential roles in this type of networks [15–18]. These distribution properties of our network are similar to other biological networks previously reported [15–17].

### 2.2. HCMV Entry Activates a Complex Systemic Network

**Figure 2:**
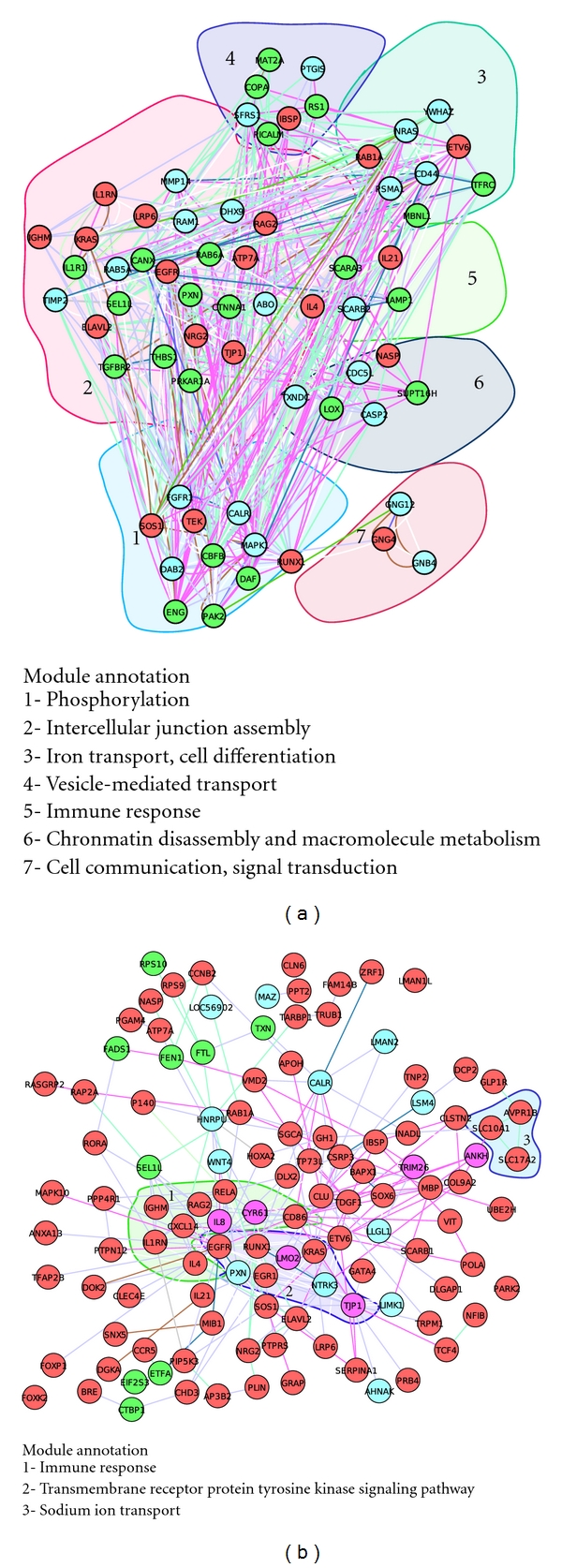
Systemic networks and functional modules activated by HCMV attachment and entry. The complete network activated by HCMV entry is listed in Supplementary Tables S2 and S3. Only parts of entire networks are shown for clarity. (a) Functional modules activated at 5 min PI; (b), activated network at 25 min PI.

After constructing the comprehensive network database, we next enriched the network (Figure 1) with genes significantly altered by HCMV attachment and entry. Genes were extracted from genomewide transcriptome significantly altered by HCMV infection at 5 min and 25 min, respectively of human primary foreskin fibroblasts, a common cell line used as a model of HCMV infection (Section 4). A total of 408 and 240 genes were obtained at 5 min and 25 min PI, respectively (Supplementary Table S2-S3). The enriched network became a systemic network activated by HCMV attachment and entry, and it was further decomposed into functional modules in basis of network topology and gene functions (Section 4). A total of 7 functional modules (Figure 2) were activated at 5 min PI, including phosphorylation, intercellular junction assembly, iron transport, cell differentiation, vesicle-mediated transport, immune response, chromatin disassembly and macromolecule metabolism, cell communication, and signal transduction. At 25 min PI, 3 functional modules were activated, including immune response, transmembrane receptor protein tyrosine kinase signaling pathway, and sodium ion transport. While modules of receptor system, ion transport, and immune response dominated the entire process of HCMV entry, cell adhesion and skeletal movement were featured at 5 min PI and immune response predominated in the network at 25 min PI (Figure 2). 

This rapid decrease in the number of activated genes from 408 (5 min PI) to 240 (25 min PI) (Supplementary Table S2-S3) within a very short time interval after HCMV infection, and the decline in network modules (from 7 to 3) fit the normal model of early cellular response to infection [6], in which activation of cellular signaling peaks immediately in response to infection, then rapidly declines dramatically.

### 2.3. A Systemic Upregulated Network Involved in HCMV Entry

**Figure 3:**
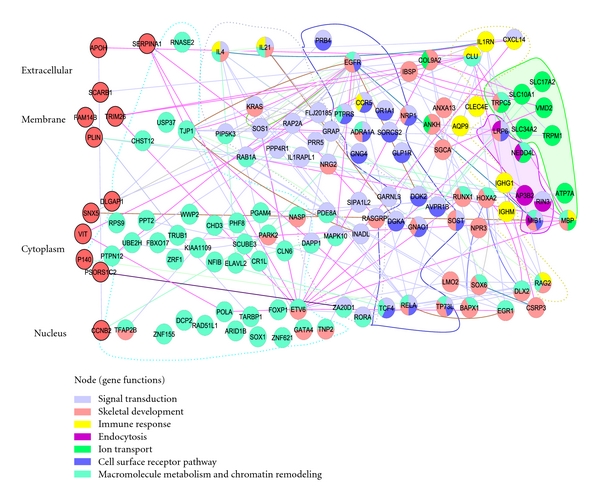
Network upregulated by HCMV entry. Genes shown here were upregulated by HCMV entry at both 5 min and 25 min PI. Genes are clustered into functional groups and color-coded. Only the primary functions for each gene are indicated. Nodes with red color on the left were not able to categorize into functional groups shown here. Cellular components are shown on the left side.

Genes that are downregulated could play an important role in HCMV attachment and entry, but the key receptor system, in particular, should be upregulated during these stages of infection [7–9]. Since the network comprising downregulated genes did not have any characterized functions (Supplementary Figure S1), we focused on a systemic upregulated network containing 123 genes (Supplementary Table S4) that were extracted from Figure 2 above with upregulation at both time points (5 min and 25 min PI). This upregulated network was decomposed into 7 functional groups (*P* < .05 based on GO term enrichment), including macromolecule metabolism and chromatin remodeling, signal transduction, cell surface receptor pathway, skeletal development, immune response, endocytosis, and ion transport (Figure 3). Consistent with previous reports about HCMV entry [6, 7, 13], the network includes many known pathways and their components upregulated by HCMV entry. Such pathways include receptor- like EGFR in the receptor group, mitogen-activated protein kinase-like MAPK10 in the signaling group, components for cytoskeletal rearrangement in the skeletal group, transcription factors located in the nucleus, cytokines located in the extracellular space, and components for calcium transport in the ion transport group. 

Importantly, our network also revealed a systemic view of the HCMV-upregulated system, in which genes are clustered into multiple functional groups of varied pathways, and simultaneously performing various functions and bioprocesses during HCMV infection. For example, the upregulated pathway group contains 18 different upregulated components (EGFR, TP73L, CCR5, OR1A1, TCF4, AVPR1B, RELA, GLP1R, GNAO1, SOST, ADRA1A, GNG4, DGKA, PRB4, NRP1, DOK2, SORCS2, PTPRS), the skeletal group 14 components (DLX2, BAPX1, SGCA, LMO2, HOXA2, IBSP, COL9A2, RUNX1, EGR1, ANKH, CSRP3, ANXA13, NPR3, SOX6), and the ion transport group 8 components (SLC34A2, ATP7A, TRPM1, MBP, SLC10A1, VMD2, TRPC5, SLC17A2). 

While signal transduction for cell communication and cell adhesion dominated the upregulated network, components for macromolecule metabolism and chromatin remodeling were surprisingly the most abundant in the network. Abundantly overexpressed components for nucleic acid metabolic process (RNASE2, NASP, ZNF621, ZNF155, POLA, ARID1B, FOXP1, TARBP1, RAD51L1, DCP2, GATA4, TFAP2B, TRUB1, ETV6, NFIB) and components for chromatin remodeling (NASP, ARID1B, CHD3, and SOX1) indicated transcription activation regulated by chromatin as one of the major bioprocesses occurring in the human host during HCMV attachment. These data revealed a complex HCMV-upregulated system that comprises several functional subnetworks that are functionally dominated by signal transduction, cell adhesion, and transcription regulated by chromatin remodeling.

### 2.4. Key Proteins in the HCMV-Upregulated Network

To identify the essential components in the HCMV upregulated network, we examined the contribution of individual components to the network by knocking out single genes in silico, which produces the experimentally proved key components in the network [14]. Special attention was paid to protein components located in the extracellular space and cell membrane (Figure 3) because these components play critical roles in initiating bioprocesses during HCMV entry or serve as potential HCMV receptors. After knocking out individual genes, we calculated the alterations in the average number of neighbors, which describes the contribution of individual nodes to network connectivity, and the mean shortest path, which measures the smallest number of links between selected nodes and essentially indicates network diameter. Node knockouts in a network would decrease network connectivity. Moreover, knockout of nodes that are higher in the network hierarchy would result in greater reduction of connectivity. As for diameter, the longer the diameter, the less interconnectivity there is in the network. Knocking out a hub would increase diameter because of the loss of short paths in a network, whereas knocking out a bottleneck would decrease diameter because the network would be broken down and the long path that normally link to subnetworks would be lost [14].

**Figure 4:**
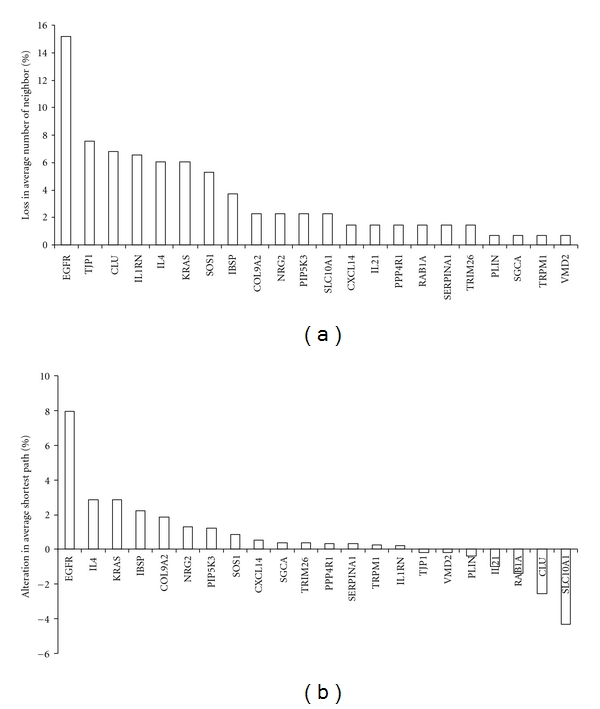
Contribution of individual genes to properties of the network enhanced by HCMV entry. Extracellular and membrane components of the network enhanced by HCMV entry (Figure 3) were individually knockedout in silico, and the effects of such knock out were calculated. Only genes with at least two direct neighbors in the network were knocked out because genes with only one direct neighbor or without neighbors are located at the end-terminal in the network and would not significantly affect the network architecture. (a) Contribution of individual genes to network connectivity. (b) Contribution of individual genes to network diameter.

The top 5 to 10% of nodes are usually considered as legitimate key hubs in this type of scale-free biological network. We selected the top 5 key genes out of 123 upregulated genes (<5%) as key genes in network. Results of in silico knock out experiments showed that the component EGFR contributed most in network connectivity and diameter (Figures 4(a) and 4(b)), indicating that it serves as a hub (highly connected proteins) in the HCMV-upregulated network. Similarly, IL4 (interleukin 4), KRAS (kirsten rat sarcoma 2 viral oncogene homolog), and IBSP (integrin-binding sialoprotein) also serve as hubs in this network activated by HCMV entry. In contrast, whereas CLU (clusterin) and SLC10A1 are also major contributors to network connectivity, knocking them out resulted in a decrease in network diameter (Figures 4(a) and 4(b)), indicating that these two components serve as bottlenecks in this network stimulated by HCMV attachment and entry.

**Figure 5:**
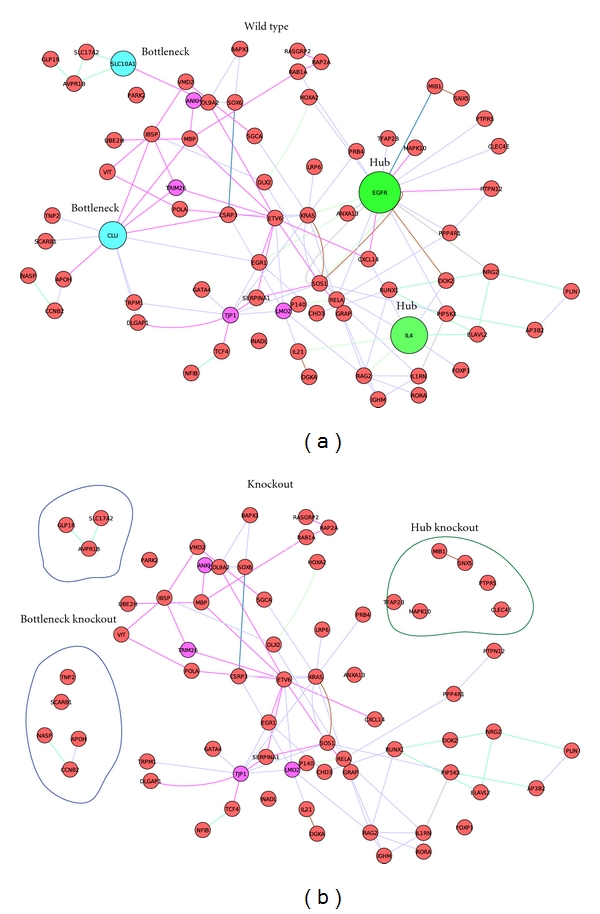
Samples of in silico gene knockout in the network enhanced by HCMV entry. (a) Entire wild-type network enhanced by HCMV entry with highlighted genes with big size to be knocked out. Blue: potential bottleneck nodes; green: potential hubs. (b) Knocking out hubs and bottlenecks alter network structure. Knocking out hubs decreases local subnetwork linkages while most of their linked neighbors would not affected. Highlighted in green circle are the consequences of knockout EGFR and IL4 with only 6 genes and 1 gene, respectively, affected by knockout, but most of genes linked to EGFR and IL4 still link to the network after knockout. Conversely, knocking out bottleneck nodes breaks down the entire network into at least two separated networks as highlighted in blue circles.

To confirm in silico the consequence of knocking out these hubs and bottlenecks, we compared the structure of mutant and wild-type network activated by HCMV attachment and entry (Figure 5). Hubs and bottlenecks are important for network and knocking them out would change the network structure, but knocking out bottlenecks would break the network into separated parts while knocking out hubs may not separate the network and may only alter linkages of local subnetwork [18]. For example, knocking out hub EGFR and IL4 leaves 6 genes and 1 gene apart from the wild-type network but most of nodes originally linked to EGFR and IL4 still link to the network although linkages of remaining nodes in the network have been changed (Figure 5(b)). Conversely, knocking out bottlenecks CLU and SLC10A1 completely breaks down the entire network into at least two independent subnetworks as highlighted in Figure 5(b). These results indicated that both potential hubs (EGFR, IL4, KRAS, and IBSP) and bottlenecks (CLU and SLC10A1) identified above play in silico important roles in the structure of the network. Further experimental data will be required to validate these key genes in vitro and in vivo, but recent evidences implicate their involvement in virus infection. For example, functions of the bottleneck gene CLU are still unclear, but recent transcriptomic and proteomic data demonstrated CLU as a top gene overexpressed by virus infection [19].

## 3. Discussion

### 3.1. HCMV Entry Triggers a Systematic Network

Studies on virus entry using traditional genetics and biochemistry approaches have identified several viral entry pathways into host cells [1, 7–9]. However, the molecular mechanisms underlying virus entry remain largely elusive. We systematically assembled the existing databases of all pathway components into a systemic scale-free network to elucidate the complexity of HCMV entry (Figure 1). The advantage of systems network approach is that it accounts for all interactions and cross-talks among components and treats the whole interactions as a network instead of linear circuits explicated by conventional approaches. The cross-talk that has been mostly ignored in conventional studies can significantly contribute to real phenotypes [20] and they were included in the present systemic network. The network constructed in the present study is based on current database. Future database updates and systemwide protein data may slightly change the linkages in our network; moreover, our network data need to be verified by direct experimental evidences like those in systems biology approaches. However, the overall architecture of our network database is not expected to change significantly because of its stable universal features and scale-free and hierarchical structure (Figure 1). Therefore, the network constructed in this study can be adapted to analyze molecular mechanisms of host-microbe interactions in general and can potentially find application in drug discovery against virus entry.

Entry of infectious agents into host cells activates complex bioprocesses [1, 21–23]. Previous studies demonstrated that HCMV entry stimulates gene expression of various pathway components, such as those involved in immune response, calcium transport, and signal transduction [6–9, 13, 24, 25]. In the present study, we systematically identified a systemic network and dynamic molecular modules activated by HCMV entry, which includes not only genes and pathways previously reported but also those uncovered in the present study (Figure 2). The dynamic activations of functional modules such as cell adhesion and skeletal movement immediately after infection (~5 min PI, Figure 2) and incoherently regulated genes in most of the modules (Figure 2) suggest that a greater complex molecular system than thought is triggered to cope with HCMV early entry.

### 3.2. HCMV Entry Requires Coordinated Network Module Interactions

Infectious agents can easily bind to cell surfaces via chemical interactions, but with low affinity. Microbe-specific receptors and coreceptors are required to strengthen these bindings, but they are not likely sufficient for a successful entry, which require subtle contributions from other functional groups. For instance, calcium transport and cytoskeletal movement, which are often observed during microbe entry, are essential for surviving some receptor-ligand interactions and play crucial roles in strengthening microbe-attachment to cell surface [26]. Similar roles are true for signal transduction, immune response, and chromatin remodeling [26]. Therefore, a highly coordinated complex network is required for microbe entry into cells but has not been elucidated until now [1, 22, 23, 27]. Here, our data revealed an HCMV-upregulated network that includes macromolecular metabolism and chromatin remodeling, signal transduction, skeletal development, immune response, endocytosis, and ion transport (Figure 3). Since this network contains all pathway components known to date to be related to HCMV entry, this network probably represents a complete coordinated network sufficient to mediate HCMV entry. Surprisingly, genes associated with nucleic acid metabolism and chromatin remodeling predominated HCMV upregulated network, suggesting that cellular nucleic activity shift is a major event during HCMV entry. Consistently, studies demonstrated that HCMV assembles at early entry its chromatin via activating cellular chromatin system [28]. It would be interesting to see more insights on cellular chromatin remodeling after HCMV infection by measuring them via high-throughput sequencing technology like CHIP-seq. 

Different microbe species utilize similar bioprocesses for entry, but pathway components mediating these bioprocesses usually exhibit species-specific. Particularly, cellular receptors are highly species-dependent. As for HCMV, integrin facilitates HCMV entry [7, 10, 13]. Indeed, a successful integrin-ligand high affinity attachment depends on how molecules underneath the membrane surface respond to integrin-ligand adhesion [26]. Other proteins, such as focal adhesion kinase, phosphatidylinositol phosphate kinase, and F-actin, need to be activated before integrin receptor activation [26, 29]. Overexpression of genes in the receptor and signal transduction groups (Figure 3) might account for the integrin activation. For example, PIP5K3 (phosphatidylinositol-3-phosphate/phosphatidylinositol 5-kinase, type I) regulates actin cytoskeleton and focal adhesion; Dok2 (docking protein 2) plays a crucial role in integrin outside-in signaling through a physical and functional interaction with integrin avb3; MAPK10 (mitogen-activated protein kinase 10, MAP kinase activity) plays a key role in focal adhesion; RAP2A (RAS related protein 2a) engages beta2 integrins; IBSP (integrin-binding sialoprotein) interacts with integrins for cell adhesion. These findings further argue for integrins receptor network for HCMV entry.

Multiple receptors have been proposed for HCMV entry, but they have not been unambiguously identified [6–9, 13]. Some of the 18 members of the receptor group in Figure 3 likely act as HCMV receptors. In particular, genes important for HCMV-upregulated network might be crucial for HCMV entry. Generally, hubs and bottlenecks are likely essential in a network [15–18]. By knocking out genes in silico, we identified EGFR, IL4, KRAS, and IBSP as hubs and CLU and SLC10A1 as bottlenecks in the HCMV upregulated network (Figures 4 and 5). Hubs and bottlenecks are new emerging concepts, and there is no available standard algorithm to identify them so far. Identifications of hubs and bottleneck may be biased depending on the algorithm and network resources used to construct the network. We merged all databases in our study (Figure 1) to eliminate database bias, and the node contributions for both network connectivity and diameter calculated here (Figures 4 and 5) were consistent with those for network centrality [30] that are essential for a network (data not shown). Key nodes identified by this approach have been proved to be true by experimental data in our previous study [14]. Therefore, the hubs and bottlenecks identified here are likely essential in the natural HCMV-upregulated network and constitute the group of proteins that are likely essential for HCMV entry. 

As a member of hubs, EGFR was previously reported as an essential component of the HCMV-upregulated network although this result needs to be confirmed [8, 9]. More detailed attention should be paid to the annotation of EGFR used in studies because there are three annotated *egfr *genes in the human genome, namely, accession number #AF277897 (located in chr7: 55,200,539-55,203,821), #U95089 (chr7: 55,054,067-55,192,136), and #U48722 (chr7: 55,054,221-55,192,136). Correspondingly, there are three probe-sets in the Affymetrix chip: 1565484_x_at, 210984_x_at, and 211607_x_at. In our gene expression experiments, expression of the *egfr* gene corresponding to accession #AF277897 was upregulated, but the other two *egfr* genes were downregulated. We focused on the EGFR with accession #AF277897 because its expression was enhanced at both time points (5 min and 25 min PI). Our network data also showed that the same EGFR likely plays an important, if not essential, role in HCMV attachment and entry, at least at the early stage (Figures 4 and 5). 

A similar role was found for the other hubs. KRAS is a protein in the small GTPase superfamily that is activated by integrins during virus entry. KRAS also interacts with multiple immune receptors and is involved in multiple pathways related to cell adhesion and virus entry, such as regulation of actin cytoskeleton, tight junction, EGFR-ErbB (erythroblastoma viral gene product homolog) signaling pathway, and MAPK signaling (http://www.genome.jp/kegg/). IL4 is a cytokine that facilitates virus entry [31]. IBSP is a sialoprotein that could bind to integrin as another component in the HCMV receptor system [7, 10]. Two glycoproteins (SLC10A1, CLU) were identified as bottlenecks (Figures 4 and 5). SLC10A1 (solute carrier family 10) belongs to sodium/bile acid cotransporter family. Ion transport plays an important role in integrin binding during virus entry as discussed above. In addition, SLC10A1 is also involved in lipid and lipoprotein metabolism (http://www.reactome.org/cgi-bin/eventbrowser?DB=gk_current&ID=73923), which might be related to the lipid rafts that signal during virus entry. CLU (clusterin) is one of the sulphated glycoproteins that is activated by virus infection [32] and regulates cell communication and signal transduction related to infection like the lectin-induced complement pathway (http://www.invitrogen.com/content.cfm?pageid=10878), and the NF-kappaB pathway [33]. Thus, these hubs and bottlenecks identified here are likely important for HCMV entry although they have to be validated by biological experiments.

Platelet-derived growth factor-alpha receptor (PDGFR*α*) was reported as a receptor for HCMV entry [12], but our data did not identified it as a key gene here, similar to previously published data [34] in which PDGFR*α* (1731_at, M21574) changed with fold of −1.1 at 30 min. The reasons for this disparity are still unclear, but the different cell lines may contribute to this difference because viral entry pathway components may vary with cells [35]. In our lab and others [34] human foreskin fibroblasts (HFFs), a frequently employed cell line for HCMV infection study, were used but the above report [12] used human embryonic lung fibroblasts (HELs). Further experiments will help clarify the conservation extent of PDGFR*α* in HCMV entry and uncover the exact roles of the key proteins identified here for HCMV entry as well as to identify more key proteins for HCMV entry. 

In this study, we used gene expression data to enrich the protein-interaction network. This activated network may not be completely consistent with those derived from protein level data, but genomics data measured by the Affymetrix microarray employed here are generally overlapping with the proteomics data [36]. Our findings about the complex network activated by HCMV entry and the HCMV upregulated network should emphasize the molecular complexity of virus entry. Targeting one or two receptor proteins as currently employed may not efficiently block virus entry and prevent virus spread across cells. The rapid change in dynamic modules and the divergence of HCMV genomes [3] make it challenging to develop an efficient strategy to block virus entry, but the upregulated network identified here and the approach we have developed should lay a framework to further dissect the molecular complexity of virus entry and facilitate efficient drug development.

## 4. Methods and Materials

### 4.1. Virus and Cells

All experiments were done using primary human foreskin fibroblasts (HFFs) (CC-2509) from Clonetics (San Diego, CA) as previously described [13]. Briefly, HFFs were cultured in a humidified incubator at 37°C in the presence of 5% CO2 and were maintained in Dulbecco's modified Eagle medium (DMEM) supplemented with 10% (vol/vol) fetal bovine serum (GIBCO/BRL), 1% (vol/vol) penicillin-streptomycin (GIBCO/BRL), and 0.2% (vol/vol) fungizone amphotericin B (GIBCO/BRL). The HCMV Towne strain obtained from the American Type Culture Collection (ATCC, Rockville, MD) was propagated in HEFs. The HCMV was harvested and purified with centrifugation, followed by a sucrose gradient centrifugation as previously described [37]. Virus stock aliquots were stored in liquid nitrogen. For the virus entry experiment, cells were grown to confluence and were infected with HCMV in normal culture medium without FBS at a MOI of 10 to ensure infection of every cell. At the indicated time point after infection (5 min and 25 min), the cells were washed once with PBS, trypsinized and collected by centrifugation. Samples treated without the virus were used as controls and processed under the identical conditions as samples treated with HCMV.

### 4.2. RNA Extraction and Microarray Hybridization

RNA was purified using the RNeasy RNA purification kit (QIAGEN Inc. Valencia, CA) followed by DNase treatment to eliminate all traces of DNA, according to the manufacturer's recommendation. GeneChip One-Cycle Target Labeling and Control Reagents (Affymetrix, Santa Clara, CA) were used to process RNA and for hybridization following the manufacturer's protocols. Affymetrix Human Genome U133 Plus 2.0 Arrays, which contains over 47,000 transcripts that completely cover the entire human genome, were employed in this study. Real time qRT-PCR was used to validate the microarray data for 11 genes and the results showed high correlation (Pearson *R* = 0.89) between them (Figure S2).

### 4.3. Network Assembly

We constructed a molecular interaction network by combining the existing network databases following the approach adopted by our previous report [14] and other publications [38, 39]. Briefly we searched the sources, targets, and interaction types from databases and then merged them together (Supplementary Table S1, e.g.). Our current network included following database, proteins, and interactions from BIND (http://bond.unleashedinformatics.com/Action), DIP (http://dip.doe-mbi.ucla.edu/), HPRD (http://hprd.org/), PreBIND http://www.blueprint.org/products/prebind/index.html), curated inflammatory disease database, EMBL human database [38–41], biocarta (http://www.biocarta.com/pathfiles/h_inflamPathway.asp, http://www.biocarta.com/pathfiles/h_LairPathway.asp) KEGG (http://www.genome.jp/kegg/pathway.html), cytokine database (http://cytokine.medic.kumamoto-u.ac.jp/), NF-*κ*B (http://people.bu.edu/gilmore/nf-kb/), and NCBI (http://www.ncbi.nlm.nih.gov). The interactions extracted from the above database were shown in Figure 1. For example, cooccurrence and literature interactions were extracted from EMBL human database and literature mining, respectively [38–41].

### 4.4. Network Analysis

The microarray data were analyzed using our previous approach [14]. Briefly, Bioconductor in R Project [42] was used for quality assessment, the background adjustment and normalization, and the gene expression values estimation. The differential expression of genes was then evaluated for infection/mock control at two time points by the two-tailed *t*-test as implemented in the limma package. Genes with *P*-values <.05 and fold change >2 between infection and control were considered as significance altered by infection (Supplementary Tables S1–S3). 

Genes with significant alterations in gene expression were used to overlap components in the protein-interaction network as previously described. These overlapped networks became the networks activated (up- and downregulated) during HCMV entry. The activated networks were decomposed into functional modules based on topological interconnection intensity (Degree cutoff >2, node score cutoff >0.2, k-score >2, max.depth >100) and gene function enrichment (*P* < .05) (http://www.geneontology.org/) [43–46]. Genes were classified according to the gene ontology database (*P* < .05) (http://www.geneontology.org/) [47].

## Supplementary Material

Figure S1. Network with down-regulated genes at both 5 min and 25 min post HCMV infection.
Figure S2. Correlation of gene expression alterations measured by real time RTPCR and microarray.
Table S1. Samples of protein interaction codes for Figure 1.
Table S2. Genes activated by HCMV at 5 min.
Table S3. Genes activated by HCMV at 25 min.
Table S4. Genes enhanced by HCMV infection at both 5 min and 25 min.
