# Supplementary material for: A Systemic Network Triggered by Human Cytomegalovirus Entry
Source: Adv Virol. 2011 May 15;2011:262080. doi: 10.1155/2011/262080 (PMC3263853; doi:10.1155/2011/262080)
Supplement: Supplementary file 1 — Figure S1. Network with down-regulated genes at both 5 min and 25 min post HCMV infection. Figure S2. Correlation of gene expression alterations measured by real time RTPCR and microarray. Table S1. Samples of protein interaction codes for Figure 1. Table S2. Genes activated by HCMV at 5 min. Table S3. Genes activated by HCMV at 25 min. Table S4. Genes enhanced by HCMV infection at both 5 min and 25 min. [file 262080.f1.pdf]

Figure S1, Network with down-regulated genes at both 5min and 25min post HCMV infection. More green of node color, more in down-regulation. No significant function can be annotated for this network in basis of gene ontology database.

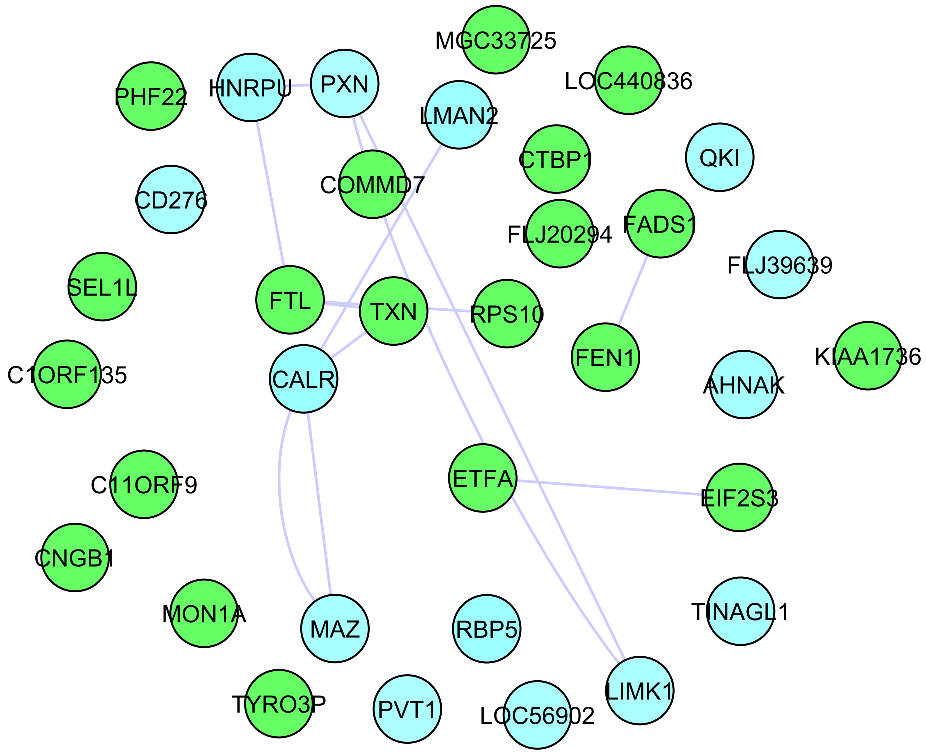

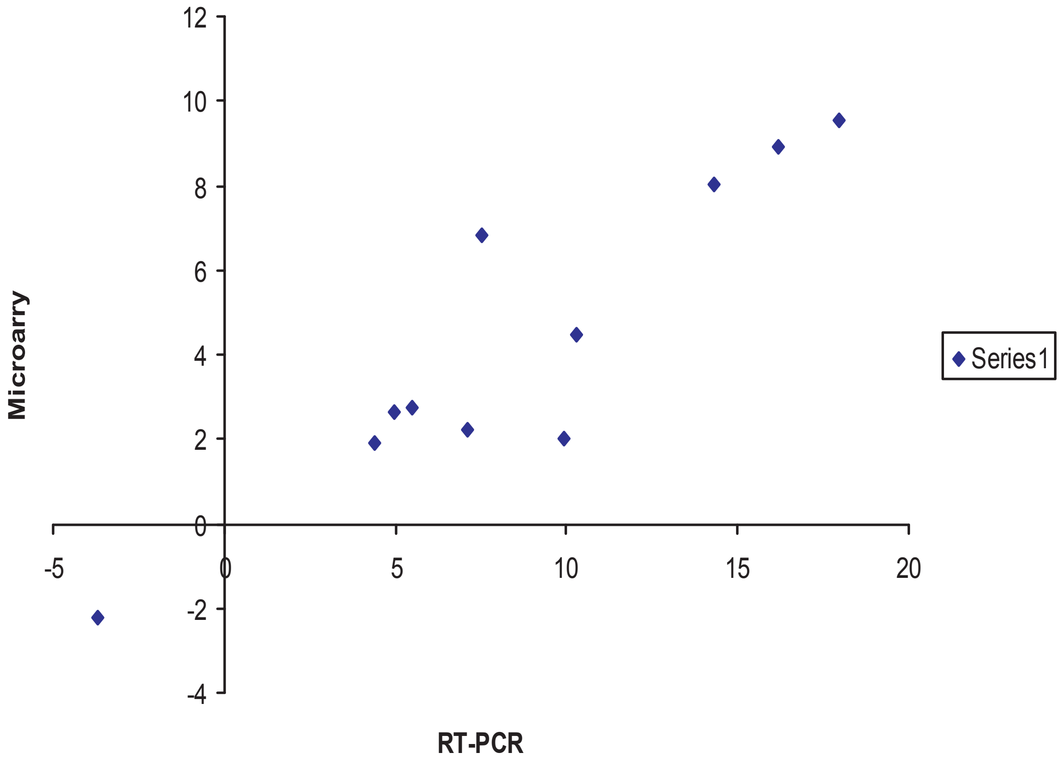

Figure S2. Correlation of gene expression alterations measured by real time RT-PCR and microarray. Total 11 genes were selected and Pearson correlation coefficient of data from these two methods was 0.89

Table S1, samples of protein interaction codes for figure 1

source	interaction type*	target
ZSWIM2	CE	TRIM42
SH3BP1	LI-ME	ARHGAP1
SH3BP1	LI-LO	AKT1
SH3BP1	LI-ME	CDC42
SH3BP1	LI-LO	PAK2
SH3BP1	LI-LO	RAC1
SH3BP1	HO	ARHGAP1
SH3BP1	EX	RAC1
SH3BP1	EX	GRB2
TMPO	PP	FOXC1
TMPO	LI-ME	TPR
TMPO	PP	TEK
TMPO	PP	ACP5
TMPO	PP	C1QTNF1
TMPO	PP	QPCT
TMPO	PP	ELAVL2
TMPO	PP	PRKAR1A

Interaction types are corresponding to that listed in figure 1.
PP represents protein-protein interaction
CE for Coexpression
LI-ME for literature-medium
LI-LO for literature-Low
HO for homology
EX for experimental
Literature-low,AG, medium, high respectively with max match 10,5,3,1.
All literature searches use context, concept lexicon restricts and interaction lexicon
Interacion resources were extracted from databases listed in materials and methods

Table S2 Genes activated by HCMV at 5min

ID	25min fold	5min fold change
KIAA0947	1.85	2.04
SHC1	-1.49	-2.2
PPT2	10.62	10.32
TARBP1	3.38	3.72
CNGB1	-5.28	-6.44
PVT1	-2.01	-7.24
DAPP1	2.49	3.92
FLJ35205	1.6	2.04
KIAA1736	-2.52	-2.08
CBFB	-1.71	-4.52
COL9A2	2.68	2.74
MGC3372[illegible]	-2.52	-2.08
RASGRP2	2.41	2.21
ADRA1A	6.29	8.14
GLP1R	2.43	3.12
LOC28350	1.74	3.63
PAFAH1B[illegible]	-1.23	-2.45
TMEM30A	-1.32	-3.04
SELO	2.14	2.61
TMEM49	1.54	2.24
SCARA3	-1.25	-3.83
IL4	5.3	4.7
CASP2	-1.52	-2.38
CLIC4	-1.44	-2.49
CPD	-1.2	-2.55
RAB1A	2.52	3.06
EDG3	-1.79	-4.04
AMFR	-1.6	-3.42
FEN1	-3.8	-4.82
RNASE2	6.1	6.59
GLUL	-1.38	-2.06
SOS1	5.13	7.92
ETFA	-14.89	-12.77
PRO1483	2.07	2.25
CDC5L	-1.63	-2.03
TJP1	2.01	3.32
LOC40083	1.6	2.04
C11ORF9	-3.8	-4.82
SOX6	2.34	2.86
PAK2	-1.44	-2.61
MTNR1A	1.88	2.33
FGFR1	-1.46	-2.18
FRMD1	2.36	2.47
ENG	-1.7	-3.81
FTL	-3.45	-2.78
HNRPU	-2.35	-4.73
CRKRS	-1.5	4.85

CALM2	-1.55	-2.61
ARHGDIA	-1.19	-2.12
RS1	-1.76	-4.47
DOK2	7.89	9.84
TEX14	-1.2	-6.95
SEL1L	-2.55	-2.52
PGAM4	6.09	13.62
PHF8	2.48	2.18
SOX1	4.58	3.38
C12ORF38	-1.64	-2.37
ELL2	-1.21	-2.35
CT45-1	2.44	3.32
DAAM2	-1.76	-2.02
LOC50351	2.83	4.02
RAB6A	-1.53	-4.54
RAG2	3.52	4.46
LOC28455	2.4	10.13
NRG2	3.63	3.71
DKFZP781	1.85	2.04
MGC13821	-1.02	6.28
CYBRD1	-1.33	-3.62
TINAGL1	-2.16	-2.39
LOX	-1.55	-3.82
SGCA	57.8	49.03
ZNF155	9.43	8.79
HP1BP3	-1.78	-2.09
PSMA1	-1.31	-2.12
FLJ45455	-1.61	-2.67
ZNF43	-1.74	-2.37
SCUBE3	2.05	2.48
KCNC3	1.95	14.88
SLC7A8	-1.08	-2.05
MYH16	-1.56	-2.26
TMED2	-1.2	-2.24
MGC13187	2.14	2.61
ARL6IP2	-1.67	-2.35
RASGRF2	1.98	2.02
PXN	-2.02	-4.05
ABCC4	-1.59	-2.55
CHMP4B	-1.53	-2.14
NSMAF	-1.24	-3.93
FLJ40244	-1.02	6.28
MGC10424	3.22	4.92
PCDHGA1	-1.44	-4
PPP1R7	2.8	2.82
TXN	-2.86	-2.06
TRPC5	3.46	4.28
APOH	4.24	4.76
BAPX1	3.74	4.95
AVPR1B	2.4	3.13
MMP14	-1.58	-2.03

IL1R1	-1.43	-5.99
TYRO3P	-14.89	-12.77
FUS	-1.72	-2.05
MIB1	3.17	5.57
SCARB2	-1.44	-2.28
RNF170	3.66	2.96
FADS1	-3.8	-4.82
ATP13A3	-1.39	-2.3
PRKAR1A	-1.85	-3.3
SUV420H2	-1.48	-2.52
IBSP	2.25	3.47
RPS9	2.21	2.98
LOC38879	2.58	2.56
ASPH	-1.59	-3.37
YWHAZ	-1.37	-2.25
WDR1	-1.44	-2.81
SMAD3	-1.57	-2.66
YTHDF3	-1.27	-2.97
MBP	2.12	2.1
CD209	1.56	2.76
ZADH2	-1.51	-2.28
SORBS3	-1.76	-2.39
DAF	-1.83	-2.65
RUNX1	3.95	4.98
LRP6	2.6	2.82
EHD2	-1.51	-3.38
ANXA13	2.68	3.43
UBE2H	2.94	2.92
TRAM1	-1.21	-2.1
P140	2.81	2.31
CANX	-1.7	-2.52
CYB5R3	-1.23	-2.29
PCDHGB3	-1.44	-4
IL1RAPL1	2.17	2.61
KIAA0157	1.71	2.11
ZA20D1	3.26	3.51
FBXO17	2.1	2.02
BRUNOL5	2.17	3.89
CALM1	-1.55	-2.61
TRIM26	2.01	2.16
NMNAT3	2.52	2.89
VMD2	2.79	2.97
AXL	1.57	2.36
LOC40153	2.79	3.42
NPR3	23.94	31.61
ARPC4	-1.67	-2.22
RAP2A	2.44	2.59
MYO1G	2.02	3.14
LRRFIP1	-1.35	-2.36
EGR1	2.36	2.38
FAM14B	2.37	2.11

ELAVL2	3.26	4.27
LMO2	2.07	2.25
PHF22	-5.03	-6.18
KIAA1257	2.65	3.19
CXCL14	3.57	5.54
CSRP3	2.44	2.92
DKFZP434	3.23	4.85
MBNL1	-1.29	-3.67
LMAN1	-1.22	-2.16
MYH14	2.61	3.06
C18ORF4	-1.38	-2.41
LOC38845	3.06	3.49
LOC56902	-2.09	-3.07
EP400	1.92	2.43
PCDHGB5	-1.44	-4
NRAS	-1.2	-2.26
YKT6	-1.74	-2.26
LOC38965	2.33	2.81
POLR2D	1.75	2.31
GNG12	-1.17	-2.29
GPX2	2.11	2.25
C12ORF40	1.72	2.63
LOC33931	1.63	2.38
LOC28553	2.47	2.47
FLJ90524	1.86	2.19
LAMP1	-1.91	-2.56
OTOA	3.92	2.96
SFTPB	1.61	2.49
VIT	2.81	2.84
RBP5	-2.4	-7.87
GRAP	3.12	2.86
EPHA8	-1.71	-9.34
CCR5	3.4	4.17
CD52	1.76	2.21
IL6ST	-1.67	-7.95
C20ORF10	2.58	2.56
ZRF1	2.18	2.94
LMAN2	-2.43	-2.03
LMAN1L	4.7	2.34
DHX9	-1.27	-2.28
AP3B2	4.95	4.6
ARPP-21	8.94	8.92
CYP4F12	1.95	2.03
PDE8A	3.46	3.87
LOC44105	-1.74	-2.62
DKFZP434	-1.57	-2.72
TFRC	-1.51	-9.09
PTPRS	3.58	3.38
PTPN12	3.75	6.04
PPP4R1	2.35	2.34
TMEM119	-1.61	-2.67

LOC90693	3.22	4.92
MAP2K2	-1.64	-2.39
SLC34A2	2.53	4
XPNPEP1	1.88	2.35
VRK3	1.93	2.95
GPM6B	2.13	2.22
DCP2	2.63	2.52
WWP2	10.45	10.67
AMOTL1	1.27	-2.04
INADL	3.86	4.87
ZNF235	2.53	4.17
TAF2	1.97	2.32
SERPINA1	3.11	3.31
C19ORF6	-1.6	-3.42
ARPP21	8.94	8.92
TRPM1	5.26	8.8
LIMK1	-2.03	-2.46
TGFBR2	-1.44	-4.15
KIAA1109	3.52	4.04
CPLX2	1.96	2.53
FLJ39639	-2.03	-2.78
PIP5K3	4.59	5
SLC17A2	2.66	2.93
SDHC	-1.42	-2.31
DGKA	2.14	2.57
STX1B2	3	2.06
PCDHGC3	-1.44	-4
PTGIS	-1.76	-2.32
LOC33879	6.58	9.65
PARK2	2.38	3.74
POLA	5.3	4.4
SEC61A1	-1.39	-2.97
RAD51L1	3.85	3.21
GNAO1	3.23	4.85
CR1L	2.21	2.28
UBXD3	-1.47	-3.66
SEZ6	2.47	3.2
LOC39170	3.27	2.78
DNAH10	3.52	2.33
MGC13175	-1.48	3.01
LOC38923	1.99	3.36
ARID1B	2.67	2.36
ADAM9	-1.36	-2.31
HSPB6	-1.43	-2.65
LOXL2	-1.32	-3.26
PLIN	4.63	3.35
MON1A	-3.45	-3.11
FN1	-1.54	-6.4
SYT2	-1.68	-2.51
STIL	2.41	2.7
HOXA2	3.93	7.3

GNB4	-1.45	-2.01
NBR1	-1.49	-2.05
TFAP2B	2.84	3.27
DDX3X	-1.32	-2.24
SIPA1L2	4.06	7.34
BTN3A1	-1.26	-2.17
RAB5A	-1.4	-2.25
CEP27	2.71	2.51
DAB2	-1.08	-2.44
TBC1D1	2.44	2.58
PSORS1C	3.03	2.79
SLC10A1	2.29	2.55
ZFP36L2	-1.65	-2.25
CD44	-1.3	-2.3
SAT	1.78	2.57
SNIP	2.81	2.31
NFIB	3.39	3.97
LOC22296	3.5	4.65
GNS	-1.38	-3.24
AQP9	2.11	2.47
COPA	-1.56	-6.58
FGFR1OP	1.24	2.32
OR1A1	2.11	2.19
ANKRD17	-1.39	-2.38
ACSS1	1.18	2.08
39327	-1.28	-2.34
GNG4	2.79	2.96
MAZ	-2.16	-2.31
AHNAK	-2.17	-12.74
CD276	-2.11	-9.3
FOXP1	2.88	2.81
ATP7A	6.09	13.62
TPP1	-1.32	-2.81
MUSTN1	2.42	2.63
THBS1	-1.69	-2.6
NNT	-1.3	-2.36
MAT2A	-1.85	-3.21
TEX13B	5.99	5.76
QKI	-2.11	-3.27
TEK	1.74	2.26
SCARB1	19.51	22.55
DBC1	3.32	3.37
STC1	-1.32	-3.36
PRRX1	-1.2	-2.39
MCL1	-1.43	-4.2
CLU	3.04	2.98
NEDD4L	38.2	88.76
LDLRAP1	1.13	4.38
RUTBC2	1.54	3.66
MPZL1	-1.35	-2.13
RIN3	2.69	2.83

RPS4Y1	1.39	2.17
ZNF621	2.37	2.39
ANKH	2.01	2.16
KIAA1199	-1.6	-2.51
PCDHGA1	-1.44	-4
EIF2S3	-2.68	-4.35
MAPK10	7.65	14.07
CCNB2	2.47	2.54
HMGA1	-1.66	-4.35
GPIAP1	-1.08	-2.03
PCDHGB1	-1.44	-4
CAPN13	1.73	3.66
CALM3	-1.44	-2.48
DCUN1D1	-1.52	-3.14
MGC13822	-1.02	6.28
NASP	3.54	4.47
MID2	5.99	5.76
TCF4	2.74	3.27
LOC14566	2.27	3.3
DLGAP1	4.05	5.31
CLEC4E	2.87	2.8
RELA	2.23	2.85
CALR	-2.5	-2.09
MAPK1	-1.5	-2.04
PICALM	-1.21	-2.57
FGF5	-1.07	-2.35
FLJ20294	-2.52	-2.08
CD109	-1.6	-2.24
GARNL3	4.84	5.57
LOC91431	3.94	3.49
IL1RN	6.81	9.21
RORA	2.9	2.03
RASD1	-1.89	-7.77
PLEC1	-1.26	-2.17
SCD	-1.46	-3.58
WNT6	-1.42	-2.89
DLX2	2.56	3.19
SFRS1	-1.21	-2.43
VAMP3	-1.12	-2.26
RGS13	-3.84	2.14
KRAS	8.36	7.47
TMEM83	3.04	3.13
NDE1	-1.6	-2.1
C1ORF135	-7.17	-4.96
ABO	-1.85	-2.07
CDV3	-1.84	-3.78
ATP11A	1.74	3.63
LOC55417	1.86	3.21
DCBLD2	-1.95	-3.19
C1ORF181	-1.76	-2.59
PCDHGA2	-1.44	-4

PRR5	2.52	2.26
COL6A1	-1.38	-2.86
SLC7A4	4.38	2.87
SOST	3.17	4.69
LENEP	1.9	3.56
IL21	3.57	4.5
TNP2	2.37	3.64
GPR176	-1.27	-2.24
CHD3	2.78	3.96
IGHM	2.23	2.69
CTNNA1	-1.63	-3.17
PRB4	4.66	6.93
CTBP1	-2.68	-2.04
ARHGAP1	-1.37	-2.28
CHST12	3.11	3.99
FLJ20185	2.52	2.26
OSMR	-1.06	-2.19
ADAMTSL	2.65	3.05
EIF4G2	-1.17	-4.43
CLN6	2.79	4.08
RPS10	-2.52	-2.08
LOC44258	-1.48	3.01
GP6	1.53	2.04
TRUB1	2.36	2.41
GTF2I	-1.41	-3.32
SPEN	1.08	-2.36
EXOC5	-1.21	-2.13
IFT172	1.85	2.14
PPFIBP1	-1.6	-2.04
ARHGAP1	-1.5	-2.83
GATA4	2.86	3.36
ATP2B4	-1.18	-2.47
EPAS1	-1.72	-5.14
EGFR	6.67	5.98
LILRB3	1.94	2.05
TXNDC	-1.28	-2.19
SNX5	2.48	2.18
APH1A	-1.84	-3.25
USP37	3.02	3.04
ETV6	4.08	4.23
NRP1	2.06	2.7
TP73L	2.22	2.29
LOC40575	3.74	4.86
YLPM1	4.28	5.18
MGC3536	2.38	2.43
SUPT16H	-1.82	-4.82
LOC44083	-3.24	-2.82
TMEM116	3.19	3.7
SORCS2	2.91	2.83
SLC39A14	-1.56	-2.35
IGHG1	2.23	2.69

TIMP2	-1.72	-2.48
ARF1	-1.9	-2.58
MGC10427	2.58	2.56
COMMD7	-57.46	-4.92

Table S3 Genes activated by HCMV at 25min

ID	25 min folc	5min fold change
PDE8A	3.46	3.87
PPT2	10.62	10.32
TARBP1	3.38	3.72
PTPRS	3.58	3.38
PTPN12	3.75	6.04
PPP4R1	2.35	2.34
LOC90693	3.22	4.92
CNGB1	-5.28	-6.44
PVT1	-2.01	-7.24
DAPP1	2.49	3.92
SLC34A2	2.53	4
GH1	2.19	1.97
GPM6B	2.13	2.22
KIAA1736	-2.52	-2.08
DCP2	2.63	2.52
WWP2	10.45	10.67
COL9A2	2.68	2.74
INADL	3.86	4.87
MGC33725	-2.52	-2.08
ZNF235	2.53	4.17
ADRA1A	6.29	8.14
RASGRP2	2.41	2.21
GLP1R	2.43	3.12
SERPINA1	3.11	3.31
ARPP21	8.94	8.92
TRPM1	5.26	8.8
SELO	2.14	2.61
LIMK1	-2.03	-2.46
KIAA1109	3.52	4.04
IL4	5.3	4.7
FLJ39639	-2.03	-2.78
PIP5K3	4.59	5
SLC17A2	2.66	2.93
RAB1A	2.52	3.06
DGKA	2.14	2.57
STX1B2	3	2.06
RNASE2	6.1	6.59
FEN1	-3.8	-4.82
ATXN2	2.07	2
LOC33879	6.58	9.65
SOS1	5.13	7.92
ETFA	-14.89	-12.77
PRO1483	2.07	2.25
TJP1	2.01	3.32
PARK2	2.38	3.74
POLA	5.3	4.4
NID1	2.17	1.43

RAD51L1	3.85	3.21
C11ORF9	-3.8	-4.82
SOX6	2.34	2.86
GNAO1	3.23	4.85
CR1L	2.21	2.28
SEZ6	2.47	3.2
FRMD1	2.36	2.47
LOC39170	3.27	2.78
FTL	-3.45	-2.78
DNAH10	3.52	2.33
HNRPU	-2.35	-4.73
ARID1B	2.67	2.36
PLIN	4.63	3.35
STIL	2.41	2.7
MON1A	-3.45	-3.11
C9ORF95	3.33	1.11
LOC28366	2.36	1.6
HOXA2	3.93	7.3
DOK2	7.89	9.84
TFAP2B	2.84	3.27
IL8	2.06	1.33
CCNL1	2.14	1.87
PRO0478	4.11	-1.8
SIPA1L2	4.06	7.34
SEL1L	-2.55	-2.52
PGAM4	6.09	13.62
CEP27	2.71	2.51
PHF8	2.48	2.18
SOX1	4.58	3.38
TBC1D1	2.44	2.58
PSORS1C	3.03	2.79
SLC10A1	2.29	2.55
CT45-1	2.44	3.32
SNIP	2.81	2.31
NFIB	3.39	3.97
NOL55	-2.96	1.14
LOC22296	3.5	4.65
LOC50351	2.83	4.02
AQP9	2.11	2.47
RAG2	3.52	4.46
LOC28455	2.4	10.13
OR1A1	2.11	2.19
NRG2	3.63	3.71
ZNF155	9.43	8.79
TINAGL1	-2.16	-2.39
SGCA	57.8	49.03
GNG4	2.79	2.96
MAZ	-2.16	-2.31
AHNAK	-2.17	-12.74
CD276	-2.11	-9.3
FOXP1	2.88	2.81

ATP7A	6.09	13.62
SCUBE3	2.05	2.48
MUSTN1	2.42	2.63
MGC1318[illegible]	2.14	2.61
LOC38872	2.18	1.95
TEX13B	5.99	5.76
QKI	-2.11	-3.27
SCARB1	19.51	22.55
PXN	-2.02	-4.05
DBC1	3.32	3.37
CLU	3.04	2.98
NEDD4L	38.2	88.76
MGC1042[illegible]	3.22	4.92
PPP1R7	2.8	2.82
RIN3	2.69	2.83
TXN	-2.86	-2.06
TRPC5	3.46	4.28
ZNF621	2.37	2.39
ANKH	2.01	2.16
BAPX1	3.74	4.95
APOH	4.24	4.76
AVPR1B	2.4	3.13
EIF2S3	-2.68	-4.35
MAPK10	7.65	14.07
CCNB2	2.47	2.54
BRE	2.57	1.74
TYRO3P	-14.89	-12.77
SLC7A13	2.2	1.44
MIB1	3.17	5.57
NASP	3.54	4.47
LOC28313	-2.5	-1.43
RNF170	3.66	2.96
MID2	5.99	5.76
FADS1	-3.8	-4.82
TCF4	2.74	3.27
DLGAP1	4.05	5.31
LOC14566	2.27	3.3
IBSP	2.25	3.47
CLEC4E	2.87	2.8
RELA	2.23	2.85
RPS9	2.21	2.98
LOC38879	2.58	2.56
CALR	-2.5	-2.09
FLJ20294	-2.52	-2.08
GARNL3	4.84	5.57
LOC91431	3.94	3.49
IL1RN	6.81	9.21
RORA	2.9	2.03
MBP	2.12	2.1
DLX2	2.56	3.19
WNT4	-2.27	-2

SC65	-2.96	1.14
RUNX1	3.95	4.98
LRP6	2.6	2.82
ANXA13	2.68	3.43
RGS13	-3.84	2.14
UBE2H	2.94	2.92
TP53INP1	2.09	1.87
P140	2.81	2.31
TMEM83	3.04	3.13
KRAS	8.36	7.47
C1ORF135	-7.17	-4.96
IL1RAPL1	2.17	2.61
ZA20D1	3.26	3.51
FBXO17	2.1	2.02
FOXK2	4.11	-1.8
MALAT1	-2.5	-1.43
BRUNOL5	2.17	3.89
TRIM26	2.01	2.16
NMNAT3	2.52	2.89
VMD2	2.79	2.97
TDGF1	12.6	-1.74
PRR5	2.52	2.26
SLC7A4	4.38	2.87
SOST	3.17	4.69
LOC40153	2.79	3.42
NPR3	23.94	31.61
CYR61	2.09	1.87
RAP2A	2.44	2.59
IL21	3.57	4.5
TNP2	2.37	3.64
MYO1G	2.02	3.14
CHD3	2.78	3.96
IGHM	2.23	2.69
PRB4	4.66	6.93
FAM14B	2.37	2.11
CTBP1	-2.68	-2.04
EGR1	2.36	2.38
ELAVL2	3.26	4.27
PHF22	-5.03	-6.18
LMO2	2.07	2.25
LSM4	-2.13	-1.55
KIAA1257	2.65	3.19
CXCL14	3.57	5.54
CSRP3	2.44	2.92
CHST12	3.11	3.99
FLJ20185	2.52	2.26
DKFZP434	3.23	4.85
ADAMTSL	2.65	3.05
MYH14	2.61	3.06
CLN6	2.79	4.08
RPS10	-2.52	-2.08

LOC38845	3.06	3.49
NTRK3	-2.43	1.56
LOC56902	-2.09	-3.07
TRUB1	2.36	2.41
LOC38965	2.33	2.81
ADAMTS1	68.77	-1.84
GATA4	2.86	3.36
CLSTN2	2.59	1.42
EGFR	6.67	5.98
SNX5	2.48	2.18
GPX2	2.11	2.25
USP37	3.02	3.04
ETV6	4.08	4.23
VPS53	2.28	2
NRP1	2.06	2.7
LOC40575	3.74	4.86
TP73L	2.22	2.29
YLPM1	4.28	5.18
CD86	2.18	1.98
LOC28553	2.47	2.47
LLGL1	-2.08	-1.97
MGC3536	2.38	2.43
OTOA	3.92	2.96
LOC44083	-3.24	-2.82
TMEM116	3.19	3.7
SORCS2	2.91	2.83
VIT	2.81	2.84
RBP5	-2.4	-7.87
GRAP	3.12	2.86
CCR5	3.4	4.17
IGHG1	2.23	2.69
C20ORF10	2.58	2.56
ZRF1	2.18	2.94
C1ORF156	3.6	1.69
LMAN2	-2.43	-2.03
LMAN1L	4.7	2.34
AP3B2	4.95	4.6
MGC10427	2.58	2.56
COMMD7	-57.46	-4.92
ARPP-21	8.94	8.92

Table S4 Genes enhanced by HCMV infection at both 5min and 25min

Genes	5min fold c	25min fold change
RAD51L1	3.21	3.85
FAM14B	2.11	2.37
AP3B2	4.6	4.95
LRP6	2.82	2.6
KIAA1109	4.04	3.52
EGR1	2.38	2.36
RNASE2	6.59	6.1
ANKH	2.16	2.01
TP73L	2.29	2.22
ETV6	4.23	4.08
IL1RN	9.21	6.81
CCR5	4.17	3.4
DLX2	3.19	2.56
OR1A1	2.19	2.11
PDE8A	3.87	3.46
ZA20D1	3.51	3.26
PPP4R1	2.34	2.35
TCF4	3.27	2.74
SCARB1	22.55	19.51
IGHG1	2.69	2.23
TRPM1	8.8	5.26
IBSP	3.47	2.25
HOXA2	7.3	3.93
AVPR1B	3.13	2.4
RELA	2.85	2.23
USP37	3.04	3.02
ARID1B	2.36	2.67
NPR3	31.61	23.94
GATA4	3.36	2.86
GLP1R	3.12	2.43
NASP	4.47	3.54
TFAP2B	3.27	2.84
PIP5K3	5	4.59
GNAO1	4.85	3.23
DLGAP1	5.31	4.05
CCNB2	2.54	2.47
SCUBE3	2.48	2.05
RASGRP2	2.21	2.41
RPS9	2.98	2.21
TJP1	3.32	2.01
UBE2H	2.92	2.94
SOST	4.69	3.17
MAPK10	14.07	7.65
NFIB	3.97	3.39
IL21	4.5	3.57
MIB1	5.57	3.17
ANXA13	3.43	2.68
TRPC5	4.28	3.46

DAPP1	3.92	2.49
NRG2	3.71	3.63
FLJ20185	2.26	2.52
SLC34A2	4	2.53
ADRA1A	8.14	6.29
SGCA	49.03	57.8
WWP2	10.67	10.45
TNP2	3.64	2.37
FOXP1	2.81	2.88
SIPA1L2	7.34	4.06
SLC10A1	2.55	2.29
CR1L	2.28	2.21
CLN6	4.08	2.79
GNG4	2.96	2.79
PARK2	3.74	2.38
RAP2A	2.59	2.44
NEDD4L	88.76	38.2
SOX1	3.38	4.58
PHF8	2.18	2.48
RAB1A	3.06	2.52
DGKA	2.57	2.14
ZRF1	2.94	2.18
EGFR	5.98	6.67
P140	2.31	2.81
CLU	2.98	3.04
PRB4	6.93	4.66
TRUB1	2.41	2.36
INADL	4.87	3.86
GRAP	2.86	3.12
PPT2	10.32	10.62
DCP2	2.52	2.63
BAPX1	4.95	3.74
NRP1	2.7	2.06
RORA	2.03	2.9
PLIN	3.35	4.63
KRAS	7.47	8.36
ELAVL2	4.27	3.26
SOS1	7.92	5.13
COL9A2	2.74	2.68
ZNF621	2.39	2.37
TRIM26	2.16	2.01
CXCL14	5.54	3.57
PRR5	2.26	2.52
APOH	4.76	4.24
IGHM	2.69	2.23
PGAM4	13.62	6.09
SOX6	2.86	2.34
DOK2	9.84	7.89
CHD3	3.96	2.78
RUNX1	4.98	3.95
POLA	4.4	5.3

ZNF155	8.79	9.43
PTPN12	6.04	3.75
LMO2	2.25	2.07
IL1RAPL1	2.61	2.17
MBP	2.1	2.12
RAG2	4.46	3.52
SNX5	2.18	2.48
ATP7A	13.62	6.09
RIN3	2.83	2.69
GARNL3	5.57	4.84
CSRP3	2.92	2.44
VMD2	2.97	2.79
PSORS1C	2.79	3.03
SORCS2	2.83	2.91
CHST12	3.99	3.11
IL4	4.7	5.3
PTPRS	3.38	3.58
AQP9	2.47	2.11
VIT	2.84	2.81
TARBP1	3.72	3.38
CLEC4E	2.8	2.87
FBXO17	2.02	2.1
SLC17A2	2.93	2.66
SERPINA1	3.31	3.11
